# Nasal burn by nitric acid

**DOI:** 10.1016/S1808-8694(15)31080-6

**Published:** 2015-10-22

**Authors:** Fernando P. Gaspar-Sobrinho, Francisco S. Nascimento Sampaio, Hélio A. Lessa

**Affiliations:** 1 M.S. In Medicine and Health Care; Substitute Professor - Department of Surgery - Medical School of the Federal University of Bahia. Mailing Address: Fernando P. G. Sobrinho - Av. Augusto Viana 40110-160 Salvador BA; 2 M.D. Otorhinolaryngologist, Substitute Professor - Department of Surgery - Medical School of the Federal University of Bahia.; 3 PhD in Surgery, Adjunct Professor - Department of Surgery - Medical School of the Federal University of Bahia.; 4 Department of Otorhinolaryngology - Prof. Edgard Santos University Hospital - Medical School of the Federal University of Bahia.

**Keywords:** nitric acid, nose, urgency, burn

## INTRODUCTION

Substances that can cause chemical burns in skin and mucosas are improperly packaged in beverages or medication containers, or even packages of harmless appearances.

## CASE PRESENTATION

**Figure 1:**
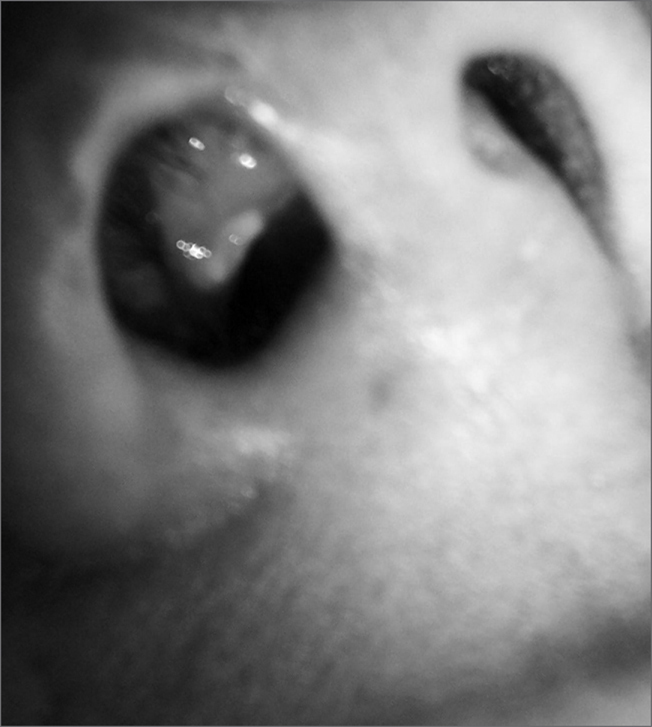
Nostril and nose vestibule with a yellow-grayish burn surrounded by erythema.

A 33 year old woman dropped in her nose a product based on nitric oxide, known as “strong water” and professionally used by her husband. The bottle with the acid was mistaken for the bottle of a topical nasal decongestant. She complained of intense pain, burning sensation, nasal obstruction and tearing. Rhinoscopy showed a burn on the nasal wing and on the nasal vestibule, of yellowish color, surrounded by erythema (Figure 1). Her nose was flushed for 15 minutes with her head down. She was then instructed to flush the affected place daily, avoid sun light exposure and use analgesic. Six months of follow up did not reveal sequelae.

## DISCUSSION

The authors did not find in the literature any case report about nitric acid being accidentally poured in the nose. Nitric acid is liquid at room temperature, and it varies between transparent and yellow, and it may even be brown or reddish in color, and bears a strong odor1. Nitric acid is used in the manufacture of pesticides, fertilizers, explosives, in the pharmaceutical industry and to clean and make metals shine.

Since there is no specific treatment for burns caused by nitric acid, the therapy advocated is support only. The toxicology service must be consulted. Steroids are used empirically in pulmonary symptoms1. We are unsure whether nasal topic steroids or systemic steroids may be of any benefit in nasal injuries.

It is possible that patients with chronic rhinitis be particularly prone to accidents like this. Population education and health care professionals' awareness are important aspects in the prevention of intoxication in rhinitis patients^2^.

## FINAL COMMENTS

Harmful substances are kept near people, without protection or warnings, and it is not uncommon that they are involved in accidents in the ear, nose and throat. Thus, the otorhinolaryngologist may be called upon to assess and treat such accidents, and such professional should also work more actively towards the prevention of such accidents in the community.
